# Supplementary material for: The master regulator OxyR orchestrates bacterial oxidative stress response genes in space and time
Source: Cell Syst. Author manuscript; Available in PMC 2025 Jun 11. (PMC7617757; doi:10.1016/j.cels.2024.10.003)
Supplement: Supplementary information [file EMS206155-supplement-Supplementary_information.pdf]

Supplementary Figures

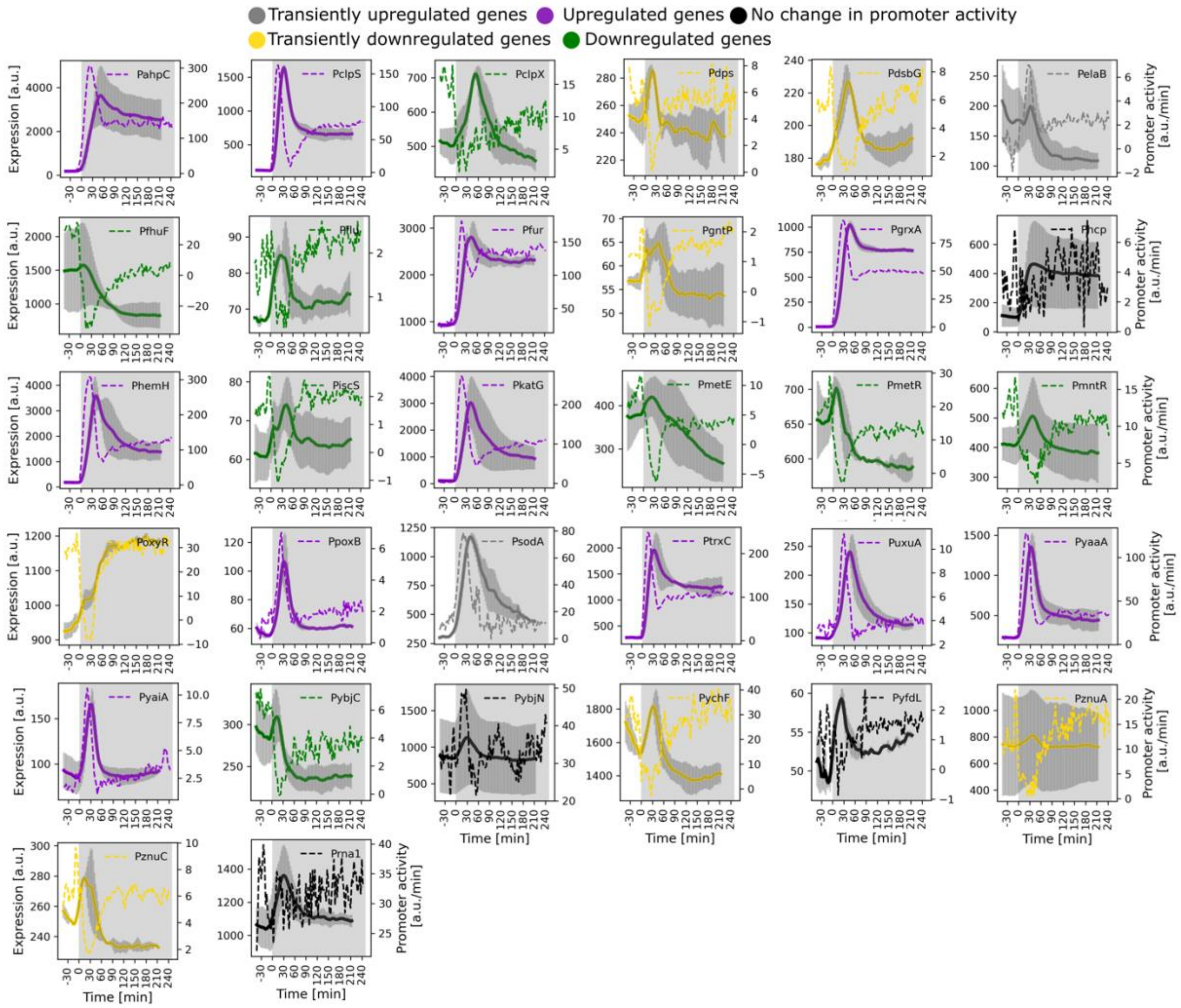

Figure S1: Gene expression dynamics during constant H_2O_2 stress

Mean expression levels (bold) and promoter activity (dash) of frontier cells for 31 transcriptional reporters (ordered alphabetically) and the constitutively expressed promoter P*rna1-mKate2* with constant 100 μM H_2O_2 treatment from t = 0 minutes (shaded region). Line colours correspond to the gene regulation categories (upregulated :purple, down-regulated: green, transiently up- (grey) or down- (yellow) regulated, no change in promoter activity: black). Error bars represent standard deviation (n ≥ 1000 cells and ≥ 2 repeats per gene).

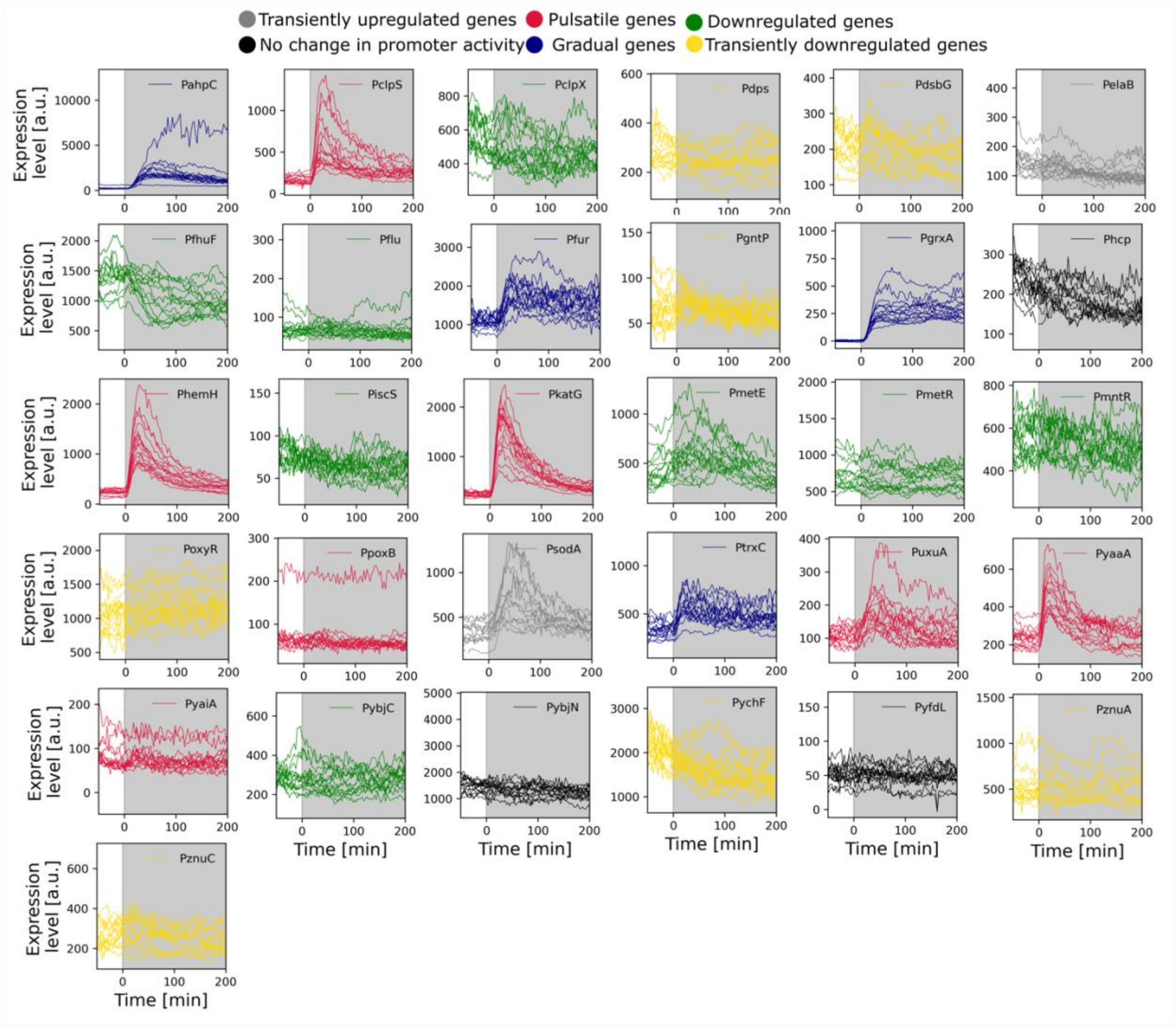

Figure S2: Single cell expression traces of oxidative stress response genes display the cell-cell variability

Single cell expression levels of mother cells for 31 transcriptional reporters with constant 100 μM H_2O_2 treatment from t = 0 minutes (shaded region). Line colours correspond to the gene regulation categories (pulsatile: pink, gradual: blue, down-regulated: green, transiently up- (grey) or down- (yellow) regulated, no change in promoter activity: black). n = 15 representative traces per gene. Note that the magnitude of the oxidative stress response is substantially lower in mother cells compared to frontier cells. Cells protect each other by actively scavenging local H_2O_2, creating steep micro-meter scale gradients of oxidative stress response. Consequently, the mother cells experience lower stress levels than frontier cells. Thus, the reduction in growth rate for mother cells is smaller, making the gene expression pulse in mother cells (Figure S2) less pronounced compared to the frontier cells (Figure S1).

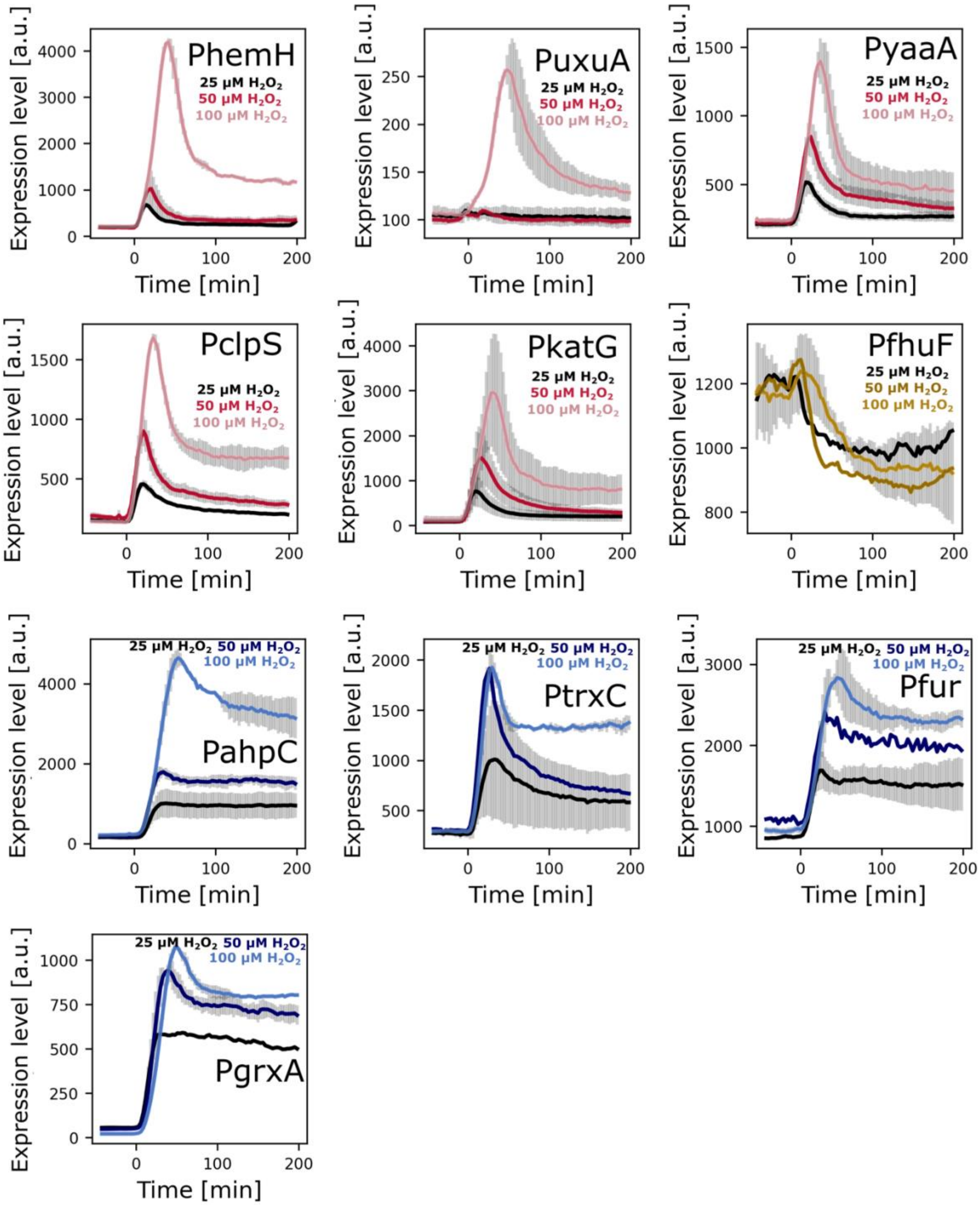

Figure S3: Pulsatile genes exhibit higher expression sensitivity to H_2O_2 stress intensity

Mean expression of frontier cells for the indicated transcriptional reporters under 25 µM, 50 µM and 100 µM H_2O_2 provided from t = 0 min (red: pulsatile, blue: gradually responding, yellow: downregulated). Error bars: standard deviation (n ≥ 2 repeats per gene).

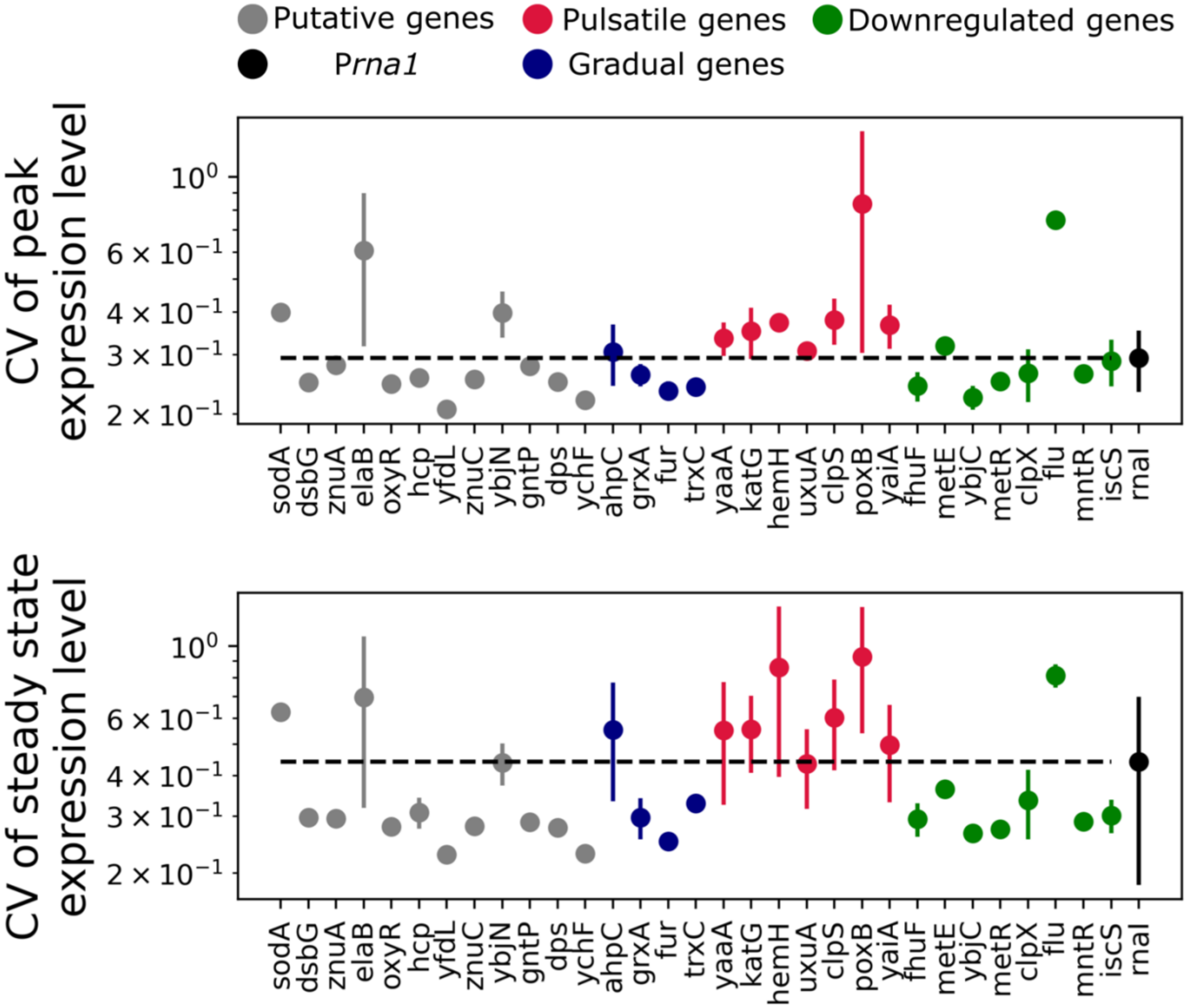

Figure S4: Cell-cell variability in gene expression magnitude for oxidative stress response genes in different regulation categories

Mean coefficient of variation for peak (top) and steady state (bottom) gene expression values for frontier cells under 100 μM H_2O_2 treatment (pulsatile: red, gradual: blue, negative: green, putative: grey, constitutive P*rna1*: black). Error bars represent standard deviation (n ≥ 3 repeats per gene)

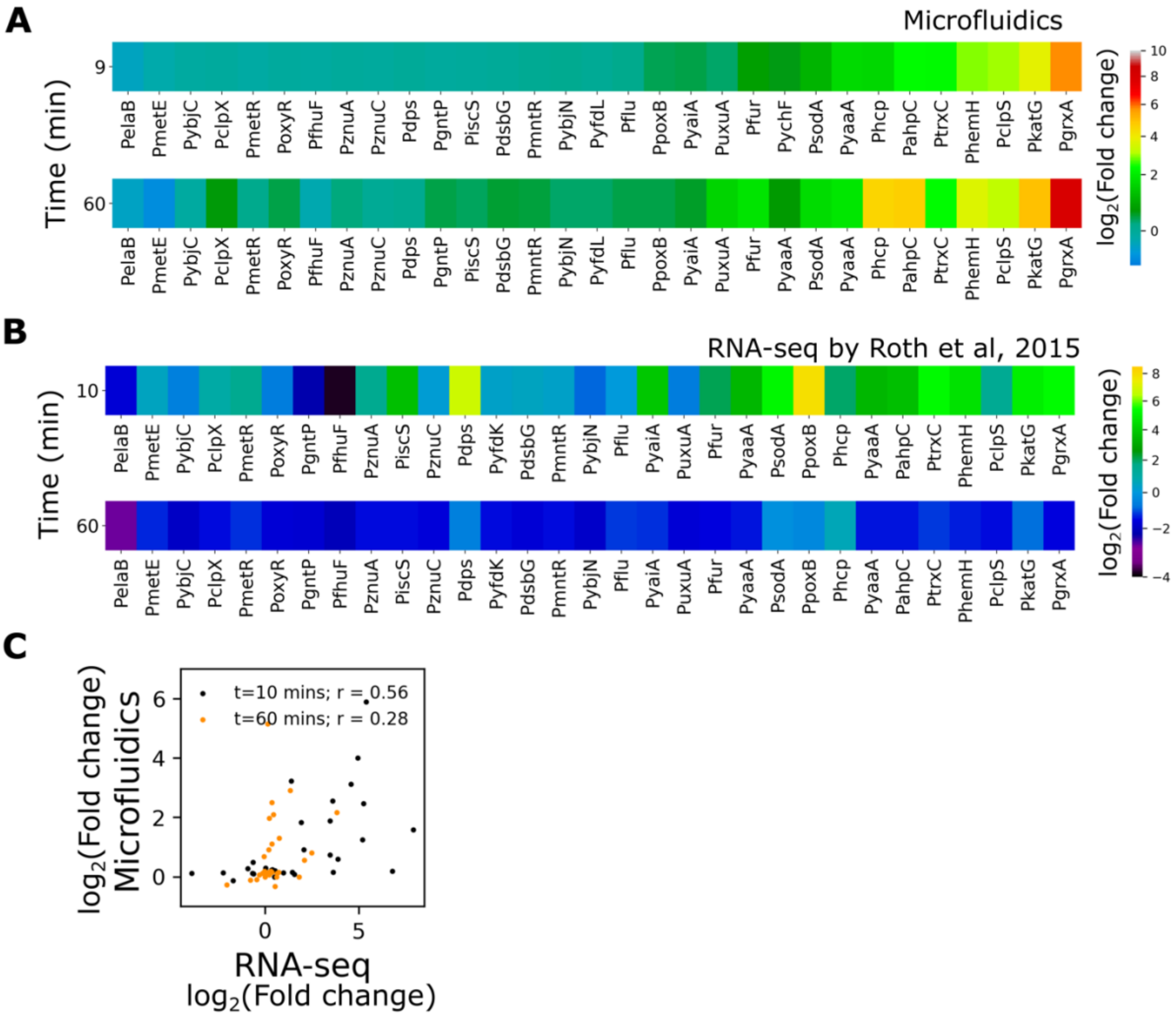

Figure S5: Gene expression level changes in our study compared to the bulk mRNA level changes, reported by Roth *et al.*, under H_2O_2 stress

(**A**) Heatmap represents mean log_2-fold change in gene expression relative to basal level of frontier cells for 31 transcriptional reporters at 9 minutes (top) and 60 minutes (bottom) post treatment with 100 μM H_2O_2 (n ≥ 1000 cells and ≥2 repeats per gene). (**B**) Heatmap represents data collected by Roth *et al*[4]. showing mean log_2-fold change in mRNA levels of cells for 31 genes at 10 mins (top) and 60 mins (bottom) after treatment with 2.5 mM H_2O_2. (**C**) Comparison of log_2-fold change in gene expression levels from microfluidic single-cell imaging and mRNA levels in panel A and B at 10 min (black) and 60 minutes (orange) post H_2O_2 treatment. Pearson's coefficient (r) for linear fit between the data sets are indicated in the plot.

Table S1: Genes chosen for this study along with their characteristics.

#	Gene	Function	Implicated in oxidative stress	Regulation in this study (peak /steady-state)	Promoter activity significance value (peak /steady-state)
1	*sodA*	Superoxide dismutase	P[47]	P/-	$1.72e^{-06}$/n.s.
2	*fhuF*	Ferric iron reductase	N[4,48,49]	N/N	$2.21e^{-18}$/$2.46e^{-10}$
3	*trxC*	Thioredoxin 2	P[4,36,44–46,48,49]	P/P	$2.83e^{-23}$/$2.65e^{-24}$
4	*yaaA*	Suppress intracellular iron levels	P[4,38,48]	P/P	$2.23e^{-09}$/$2.0e^{-12}$
5	*dps*	Fe-binding & storage	P[4,36,49,51,53]	N/-	$4.9e^{-05}$/n.s.
6	*ahpC*	Akylhydroperoxidase	P[4,36,42,43,48,49]	P/P	$1.45e^{-17}$/$4.36e^{-06}$
7	*katG*	Catalase	P[4,30,36,42,48,49]	P/P	$5.24e^{-22}$/$2.65e^{-24}$
8	*grxA*	Glutaredoxin-1	P[4,36,44,45,49]	P/P	$7.029e^{-18}$/$1.18e^{-18}$
9	*dsbG*	Sulfenic acid reductase	P[4,49,54,80]	N/-	$1.912e^{-09}$/n.s.
10	*znuA*	Zinc transport	P[36]	N/-	$7.61e^{-06}$/n.s.
11	*metE*	methionine synthase	P/N[36,55]	N/N	$3.73e^{-09}$/$2.31e^{-06}$
12	*oxyR*	OxyR	N[4,32,49]	N/-	$1.512e^{-09}$/n.s.
13	*hemH*	Ferrochelatase	P[4,41,48,49]	P/P	$7.029e^{-18}$/$1.18e^{-18}$
14	*mntR*	Mn^{2+} regulator[56]	N[4,49]	N/N	$1.359e^{-10}$/0.015
15	*gntP*	gluconate permease	N[4,48,49]	N/-	$1.512e^{-09}$/n.s.
16	*uxuA*	Mannonate hydrolase	N[4,48,49]	P/P	$3.36e^{-05}$/0.016
17	*iscS*	Iron-sulfur cluster assemby	N[40,48]	N/-	$1.29e^{-09}$/n.s.
18	*hcp*	Hybrid cluster proteins of Fe/S	P[4,49,57]	-/-	n.s./ n.s.
19	*flu*	surface adhesin	N/P[4,36,49,58]	N/N	0.0076/0.00545

20	*ybjC*	Unknown	N[4,36,48,49]	N/N	$1.39e^{-09}$/0.00036
21	*ychF*	ATPase	N[4,49,59]	N/-	$2.77e^{-06}$/n.s.
22	*yfdL*	Putative ligase	-[80]	-/-	n.s./ n.s.
23	*metR*	methionine biosynthesis[81]	P[4,36]	N/N	$6.046e^{-09}$/$3.73e^{-06}$
24	*znuC*	Zinc transport[82]	P[4,36]	N/-	$1.34e^{-06}$/n.s.
25	*ybjN*	Unknown	N[4,49]	-/-	n.s./ n.s.
26	*elaB*	Membrane integrity	P[4,60]	P/P	$2.05e^{-05}$/n.s.
27	*fur*	iron-homeostatic control protein	P[4,39,48,49]	P/P	$2.44e^{-12}$/$5.60e^{-13}$
28	*poxB*	pyruvate:quinoneoxidoreductase[83]	P[4,48]	P/P	$8.58e^{-07}$/$6.17e^{-05}$
29	*yaiA*	Unknown	P[4,48]	P/P	0.00015/0.00279
30	*clpS*	Iron sequestration	P[37]	P/P	$1.3877e^{-13}$/$1.18e^{-18}$
31	*clpX*	Iron sequestration	P[37]	N/N	$1.738e^{-05}$/$3.67e^{-06}$

P: upregulation, N: downregulation; p-values using Mann-Whitney tests where n.s. for $p>0.05$
